# Non-pterygium Escobar syndrome from compound-heterozygous *CHRNG* variants: genotype–phenotype insights

**DOI:** 10.1038/s41439-026-00340-8

**Published:** 2026-03-14

**Authors:** Jun Kido, Hiroe Ueno, Yohei Misumi, Keishin Sugawara, Suzuran Saito, Eriko Koshimizu, Naomichi Matsumoto, Mitsuharu Ueda, Kimitoshi Nakamura

**Affiliations:** 1 Department of Pediatrics, Kumamoto University Hospital, Kumamoto, Japan; 2 Department of Pediatrics, Faculty of Life Sciences, Kumamoto University, Kumamoto, Japan; 3 Department of Pediatrics, Kumamoto Takumadai Rehabilitation Hospital, Kumamoto, Japan; 4 Department of Neurology, Graduate School of Medical Sciences, Kumamoto University, Kumamoto, Japan; 5 Department of Human Genetics, Yokohama City University Graduate School of Medicine, Yokohama, Japan; 6 Department of Rare Disease Genomics, Yokohama City University Hospital, Yokohama, Japan; 7 Department of Clinical Genetics, Yokohama City University Hospital, Yokohama, Japan; 8 Medical Genome Center, National Center of Neurology and Psychiatry, Kodaira, Japan

**Keywords:** Urogenital diseases, Clinical genetics

## Abstract

Escobar syndrome is a rare congenital disorder characterized by contractures, pterygia and craniofacial anomalies. Here we report a school-age girl harboring compound-heterozygous *CHRNG* variants, NM_005199.5:c.[2T>C];[428C>G] p.[(Met1?)];[(Pro143Arg)]. She presented with neonatal asphyxia, congenital limb contractures and low-frequency hearing loss but without pterygia, maintaining normal cognition. This case underscores the phenotypic variability of *CHRNG*-related disease and alerts clinicians to recognize milder presentations that lack pterygia and to consider targeted genetic testin.

Escobar syndrome (MIM 265000) is a congenital disorder characterized by multiple pterygia (webbing), congenital contractures (arthrogryposis) and distinctive craniofacial and axial features. In the nonlethal form, survival into adulthood is possible^1^. Neurodevelopment is usually preserved. The condition is most commonly caused by autosomal recessive pathogenic variants in *CHRNG* (encoding the fetal γ-subunit of the muscle nicotinic acetylcholine receptor (AChR)) and, less frequently, by pathogenic variants in *MYH3* (encoding embryonic myosin heavy chain) or other components of the neuromuscular junction/contractile apparatus^2^.

Nicotinic AChRs include both muscle-type and neuronal-type receptors. Binding of acetylcholine to muscle-type AChRs at the neuromuscular junction induces a conformational change in the receptor’s nonselective cation pore, resulting in rapid cation influx and depolarization of the muscle membrane, thereby initiating muscle contraction^3^.

Additional variable clinical features of Escobar syndrome include intrauterine death, neonatal respiratory distress, short stature, ptosis, low-set ears and cryptorchidism in males^4^. However, the relationship between the *CHRNG* variant spectrum and clinical manifestations remains incompletely understood^5,6^.

Here, we report a female patient with compound heterozygous *CHRNG* variants, NM_005199.5:c.[2T>C];[428C>G], p.[(Met1?)];[(Pro143Arg)]. She exhibited congenital limb contractures and low-frequency hearing loss without overt pterygia. We describe her clinical course and discuss genotype–phenotype correlations in this disorder.

The patient was a female infant born at 38 weeks and 3 days of gestation, with birth measurements of length 46.0 cm (−1.2 s.d.), weight 2786 g (0.0 s.d.) and head circumference 35.0 cm (+1.5 s.d.). Apgar scores were 1 at 1 min and 6 at 5 min due to neonatal asphyxia requiring resuscitation. Neonatal jaundice was treated with phototherapy. Newborn hearing screening was reportedly normal. There was no consanguinity and no family history of neuromuscular disease.

She achieved head control at 5 months, sat independently at 8 months and walked independently at 13 months. The family noticed reduced responsiveness to sound between 1 and 2 years of age, but early otolaryngologic evaluation was inconclusive.

From birth, she showed cutaneous syndactyly of the fingers and toes (HP:0012725), joint contracture of the hand (HP:0009473) and a left ankle contracture (HP:0034677). She was comanaged by pediatric orthopedics and began ongoing physical and occupational therapy at 8 months. At school-entry screening (6 years 4 months), bilateral low-frequency hearing loss (HP:0008542) was detected, and she has since used hearing aids. Cranial computed tomography was normal.

At 8 years 4 months, the Wechsler Intelligence Scale for Children–IV showed a Full-Scale IQ of 116 (verbal comprehension 119, perceptual reasoning 106, working memory 97, processing speed 121), indicating average-to-high intellectual functioning.

At 9 years of age, her height was 122.9 cm (−1.9 s.d.) (HP:0004322) and weight was 22.0 kg (−1.1 s.d.) (Fig. 1A). Craniofacial features included hypertelorism (HP:0000316) and downslanted palpebral fissures (HP:0000494); hypertrichosis (HP:0000998) was also noted. Limb findings included adducted thumbs (HP:0001181); bilateral flexion contractures of digits 2–5 (HP:0001215); left ankle dorsiflexion (HP:0033526) limited to −5° with pes planus (HP:0001763); and a right foot with an in-toeing/varus tendency (HP:6001054). No pterygia were identified on serial examinations (Fig. 1B).

**Fig. 1:**
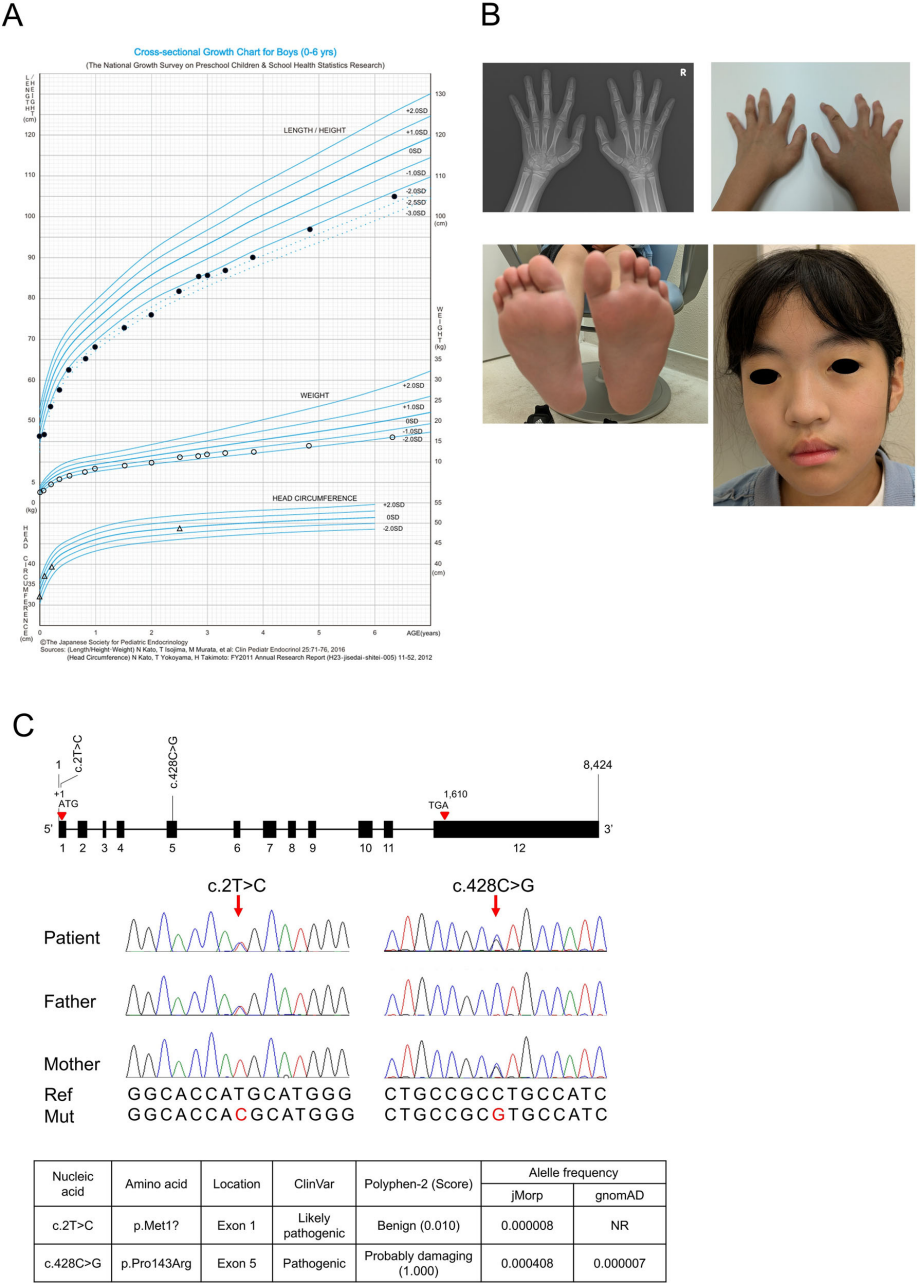
Clinical manifestations in this patient with Escobar syndrome. **A** Growth curve until age of 6 years. **B** Physical feature at age of 10 years. She developed tapered finger (HP:0001182) and mild cutaneous syndactyly (HP:0012725). Moreover, the left index finger exhibited ulnar deviation at the proximal interphalangeal joint (HP:0009487), while passive extension of the finger was preserved. There was a mild discrepancy in leg length (HP:0006388), and the formation of the medial longitudinal arches was weak, consistent with pes planus (HP:0001763). Her facial features were characterized by hypertelorism (HP:0000316), mild thick vermilion border (HP:0012471) and maxillary prognathism (HP:0000303). **C** Genetical analysis result. The c.2T>C variant was paternally inherited, and the c.428C>G variant was maternally inherited. jMorp, Japanese Multi Omics Reference Panel; NR not registered.

She was registered with the Initiative on Rare and Undiagnosed Diseases^7^, led by the Japan Agency for Medical Research and Development. Whole-exome sequencing identified compound heterozygous *CHRNG* variants, NM_005199.5:c.[2T>C];[428C>G], p.[(Met1?)];[(Pro143Arg)]. Sanger sequencing confirmed that c.2T>C was paternally inherited and c.428C>G was maternally inherited (Fig. 1C).

At 10 years and 3 months of age, she continues to receive longitudinal physical and occupational therapy, as well as podiatric and orthopedic follow-up. She demonstrates functional ambulation and full participation in school activities, with good academic performance. Laboratory evaluations, including insulin-like growth factor-1 (IGF-1), were normal (IGF-1 347 ng/ml; reference range 155–588 ng/ml) (Supplementary Data 1). Her height is 128.5 cm (−1.5 s.d.), and her weight is 28.9 kg (−0.6 s.d.).

We report a female patient with Escobar syndrome carrying compound heterozygous *CHRNG* variants, NM_005199.5:c.[2T>C];[428C>G], p.[(Met1?)];[(Pro143Arg)]. She demonstrated a relatively mild phenotype with craniofacial and limb involvement and short stature, but without pterygia. Consistent with previous reports, intellectual disability is generally not observed in this disease entity.

The variants c.2T>C p.(Met1?) and c.428C>G p.(Pro143Arg) are registered in ClinVar^8^ as ‘likely pathogenic’ and ‘pathogenic’, respectively. These variants are also listed in gnomAD^9^ and the Tohoku Medical Megabank genome database^10^. In silico analysis with PolyPhen-2 predicted c.2T>C as ‘benign’ (score 0.010, sensitivity 0.96, specificity 0.77) and c.428C>G as ‘probably damaging’ (score 1.000, sensitivity 0.00, specificity 1.00). Using ACMG/AMP criteria, we classified c.2T>C as ‘pathogenic’ (PVS1, PM2, PM3, PP4) and c.428C>G as ‘likely pathogenic’ (PS1, PM3, PP3, PP4).

We summarized previously reported pathogenic *CHRNG* variants and associated phenotypes in Table 1 and Supplementary Data 2 (refs. ^4–6,11–23^). The frequency of each clinical manifestation was evaluated using residual analysis based on contingency tables (Table 1).

**Table 1:** Relationship between clinical phenotypes and pathogenic variants.

Patient (gender, age)	Variant		Dysmorphic signs									Congenital problems			Postnatal myasthenic features		References
	Allele 1	Allele 2	Pterygia	Facial dysmorphism	Cleft palate	High-arched palate	Arthrogryposis multiplex	Arachdactyly/camptodactyly	Kyphosis/scoliosis	Short stature	Cryptorchism	Respiratory distress	Decreased movements	Facial weakness	Clinical myasthenic symptoms	Myasthenic signs on EMG	
**This study (F, 10** **y 3** **m)**	**c.2T**>**C (p.Met1?)**	**c.428C**>**G (p.Pro143Arg)**	**No**	**Yes**	**Yes**	**No**	**Yes**	**Yes**	**No**	**Yes**	**–**	**Yes (immediately after birth)**	**No**	**No**	**No**	**No**	**This study**
EG-1 II-3 (F, 12 y)*	c.13C>T (p.Gln5*)	c.13C>T (p.Gln5*)	Yes	Yes	No	Yes	Yes	Yes	Yes	Yes	–	Yes	NA	Yes	No	No	^5^
EG-1 II-5 (M, 5 y)*			Yes	Yes	No	Yes	Yes	Yes	Yes	Yes	Yes	Yes	NA	Yes	No	No	^5^
MPS044 (NA, NA)	c.55G>A (p.Gly19Arg)	c.1132_1136dup (p.Gly380Profs*39)	NA	NA	NA	NA	NA	NA	NA	NA	NA	NA	NA	NA	NA	NA	^6^
MPS038 (NA, NA)	c.56-1G>A	c.56-1G>A	NA	NA	NA	NA	NA	NA	NA	NA	NA	NA	NA	NA	NA	NA	^6^
Patient (M, at birth)	c.117dup (p.Asn40Glnfs*96)	c.117dup (p.Asn40Glnfs*96)	Yes	Yes	Yes	Yes	Yes	Yes	NA	NA	NA	Yes	NA	NA	NA	NA	^13^
MPS002 (F, NA)	c.136C>T (p.Arg46*)	c.136C>T (p.Arg46*)	Yes	Yes	NA	Yes	Yes	NA	Yes	Yes	–	NA	NA	NA	NA	NA	^4^
P9 (F, 1 y)	c.202C>T (p.Arg68*)	c.292_300dup (p.Leu100_Arg101insTrpValLeu)	Yes	Yes	NA	Yes	NA	NA	No	NA	–	NA	NA	NA	NA	NA	^14^
MPS037 (NA, NA)	c.202C>T (p.Arg68*)	c.753_754del (p.Val253Alafs*44)	NA	NA	NA	NA	NA	NA	NA	NA	NA	NA	NA	NA	NA	NA	^6^
BAB7080 (M, NA)	c.241C>T (p.Gln81*)	c.241C>T (p.Gln81*)	Yes	Yes	NA	NA	Yes	NA	NA	NA	NA	NA	NA	NA	NA	NA	^18^
**Patient 3 (M, NA)**	c.255T>A (p.Trp85*)	**c.428C**>**G (p.Pro143Arg)**	**No**	**Yes**	**NA**	**NA**	**Yes**	**Yes**	**NA**	**NA**	**Yes**	**NA**	**NA**	**NA**	**NA**	**NA**	^11^
BAB4104 (M, NA)	c.256C>T (p.Arg86Cys)	c.256C>T (p.Arg86Cys)	Yes	Yes	NA	NA	Yes	Yes	NA	NA	Yes	NA	NA	NA	NA	NA	^18^
EG-3 II-1 (M, Died at 3 m)	c.256C>T (p.Arg86Cys)	c.482G>A (p.Trp161*)	Yes	Yes	No	Yes	Yes	Yes	No	Yes	NA	Yes	Yes	Yes	No	NA	^5^
EG-4 II-1 (M, Died at 3 m)*	c.292_300dup (p.Leu100_Arg101insTrpValLeu)	c.1408C>T (p.Arg470*)	Yes	NA	NA	NA	Yes	Yes	Yes	NA	Yes	Yes	Yes	NA	NA	NA	^5^
EG-4 II-2 (M, 18 y)*			Yes	Yes	No	Yes	Yes	Yes	Yes	Yes	Yes	No	Yes	Yes	No	No	^5^
MPS001 (M, NA)	c.320T>G (p.Val107Gly)	c.320T>G (p.Val107Gly)	Yes	Yes	NA	NA	NA	NA	NA	NA	NA	NA	NA	Yes	NA	NA	^4^
MPS035 (NA, NA)	c.388del (p.Val130Cysfs*53)	c.459dup (p.Val154Serfs*24)	NA	NA	NA	NA	NA	NA	NA	NA	NA	NA	NA	NA	NA	NA	^6^
MPS031 (NA, NA)	c.397del (p.Ser133Profs*50)	c.397del (p.Ser133Profs*50)	NA	NA	NA	NA	NA	NA	NA	NA	NA	NA	NA	NA	NA	NA	^6^
MPS015 (M, 14 y)	c.401_402del (p.Pro134Argfs*34)	c.401_402del (p.Pro134Argfs*34)	Yes	NA	NA	NA	Yes	NA	NA	NA	NA	Yes	Yes	Yes	Yes	Yes	^4^
Family 3 IV:3 (M, 7 y)	c.401_402del (p.Pro134Argfs*34)	c.401_402del (p.Pro134Argfs*34)	Yes	Yes	NA	Yes	Yes	Yes	NA	NA	Yes	NA	NA	Yes	NA	NA	^20^
P5 (F, 18 y)	c.401_402del (p.Pro134Argfs*34)	c.459dup (p.Val154Serfs*24)	Yes	Yes	NA	Yes	NA	NA	Yes	NA	–	Yes	NA	NA	NA	NA	^14^
Patient 1 (M, NA)*	**c.428C**>**G (p.Pro143Arg)**	c.753_754del (p.Val253Alafs*44)	**No**	**Yes**	**NA**	**Yes**	**NA**	**Yes**	**Yes**	**NA**	**NA**	**NA**	**NA**	**NA**	**NA**	**NA**	^11^
Patient 2 (F, NA)*			**No**	**Yes**	**NA**	**NA**	**Yes**	**NA**	**NA**	**NA**	**–**	**NA**	**NA**	**NA**	**NA**	**NA**	^11^
**MPS1-1 (M, 5** **y)**	**c.428C**>**G (p.Pro143Arg)**	c.753_754del (p.Val253Alafs*44)	**NA**	**NA**	**NA**	**NA**	**Yes**	**Yes**	**Yes**	**Yes**	**NA**	**NA**	**NA**	**NA**	**NA**	**NA**	^22^
**MPS2-1 (F, 12** y)*	**c.428C**>**G (p.Pro143Arg)**	c.753_754del (p.Val253Alafs*44)	**NA**	**NA**	**NA**	**Yes**	**Yes**	**Yes**	**NA**	**Yes**	**–**	**NA**	**NA**	**NA**	**NA**	**NA**	^22^
MPS2-2 (F, NA)*			**NA**	**NA**	**NA**	**Yes**	**Yes**	**Yes**	**NA**	**Yes**	**–**	**NA**	**NA**	**NA**	**NA**	**NA**	^22^
MPS011 (F, Died at 1 d)	c.459dup (p.Val154Serfs*24)	c.459dup (p.Val154Serfs*24)	NA	NA	NA	NA	Yes	NA	NA	NA	–	Yes	NA	NA	NA	NA	^4^
Patient 1 (M, 5 y)	c.459dup (p.Val154Serfs*24)	c.459dup (p.Val154Serfs*24)	Yes	Yes	Yes	NA	Yes	NA	Yes	Yes	Yes	NA	NA	NA	NA	NA	^21^
Patient 2 (F, 13 y)	c.459dup (p.Val154Serfs*24)	c.459dup (p.Val154Serfs*24)	Yes	NA	NA	Yes	Yes	Yes	NA	Yes	–	NA	Yes	NA	NA	NA	^21^
Patient 3 (M, 4 y)*	c.459dup (p.Val154Serfs*24)	c.459dup (p.Val154Serfs*24)	Yes	Yes	NA	NA	Yes	Yes	Yes	NA	Yes	NA	NA	NA	NA	NA	^21^
Patient 4 (F, 6 y)*			NA	Yes	NA	NA	NA	NA	Yes	NA	–	Yes	NA	NA	NA	NA	^21^
JM (M, 9 d)	c.459dup (p.Val154Serfs*24)	c.459dup (p.Val154Serfs*24)	Yes	Yes	NA	NA	Yes	Yes	Yes	NA	Yes	NA	NA	NA	NA	NA	^19^
MPS033 (NA, NA)	c.459dup (p.Val154Serfs*24)	c.459dup (p.Val154Serfs*24)	NA	NA	NA	NA	NA	NA	NA	NA	NA	NA	NA	NA	NA	NA	^6^
MPS034 (NA, NA)	c.459dup (p.Val154Serfs*24)	c.459dup (p.Val154Serfs*24)	NA	NA	NA	NA	NA	NA	NA	NA	NA	NA	NA	NA	NA	NA	^6^
MPS039 (NA, NA)	c.459dup (p.Val154Serfs*24)	c.459dup (p.Val154Serfs*24)	NA	NA	NA	NA	NA	NA	NA	NA	NA	NA	NA	NA	NA	NA	^6^
P6 (F, 2 y)	c.459dup (p.Val154Serfs*24)	c.639_643del (p.Lys214Alafs*82)	Yes	Yes	NA	No	NA	NA	Yes	NA	–	NA	NA	NA	NA	NA	^14^
P2 (M, 20 y)*	c.459dup (p.Val154Serfs*24)	c.753_754del (p.Val253Alafs*44)	Yes	No	NA	Yes	NA	NA	Yes	NA	NA	NA	NA	NA	NA	NA	^14^
P3 (F, 13 y)*			Yes	No	NA	No	NA	NA	Yes	NA	–	NA	NA	NA	NA	NA	^14^
MPS042 (NA, NA)	c.459dup (p.Val154Serfs*24)	c.753_754del (p.Val253Alafs*44)	NA	NA	NA	NA	NA	NA	NA	NA	NA	NA	NA	NA	NA	NA	^6^
Patient 1 (M, 9 y)	c.459dup (p.Val154Serfs*24)	c.794T>G (p.Leu265Ser)	No	Yes	NA	Yes	Yes	NA	Yes	Yes	NA	NA	Yes	Yes	NA	NA	^23^
Family A (Fetus)	c.518dup (p.Tyr173*)	c.518dup (p.Tyr173*)	NA	NA	NA	NA	NA	NA	NA	NA	–	NA	NA	NA	NA	NA	^12^
EG-6 IV-3 (F, Died at 13 d)*	c.715C>T (p.Arg239Cys)	c.715C>T (p.Arg239Cys)	NA	Yes	No	NA	Yes	Yes	NA	NA	–	No	No	NA	NA	NA	^5^
EG-6 IV-5 (F, 7 y)*			Yes	Yes	Yes	Yes	Yes	Yes	Yes	Yes	–	No	Yes	Yes	No	No	^5^
EG-7 II-1 (F, Stillborn)	c.715C>T (p.Arg239Cys)	c.715C>T (p.Arg239Cys)	Yes	NA	NA	NA	Yes	NA	NA	NA	–	–	–	–	–	–	^5^
BAB6209 (M, NA)	c.715C>T (p.Arg239Cys)	c.715C>T (p.Arg239Cys)	Yes	Yes	NA	NA	Yes	NA	NA	NA	NA	NA	NA	NA	NA	NA	^18^
BAB7083 (F, NA)	c.715C>T (p.Arg239Cys)	c.715C>T (p.Arg239Cys)	Yes	NA	NA	NA	Yes	Yes	NA	NA	–	NA	NA	NA	NA	NA	^18^
Family 2 (Fetus)	c.715C>T (p.Arg239Cys)	c.715C>T (p.Arg239Cys)	Yes	Yes	NA	NA	Yes	NA	NA	NA	–	NA	NA	NA	NA	NA	^16^
Family 1 (Fetus)	c.753_754del (p.Val253Alafs*44)	c.753_754del (p.Val253Alafs*44)	NA	Yes	NA	Yes	Yes	NA	NA	NA	–	NA	NA	NA	NA	NA	^16^
MPS008 (M, Fetus#)	c.753_754del (p.Val253Alafs*44)	c.753_754del (p.Val253Alafs*44)	NA	Yes	NA	NA	NA	NA	Yes	NA	NA	NA	NA	NA	NA	NA	^4^
BAB6498 (M, NA)	c.753_754del (p.Val253Alafs*44)	c.753_754del (p.Val253Alafs*44)	Yes	Yes	NA	NA	Yes	NA	NA	NA	NA	NA	NA	NA	NA	NA	^18^
Family 2 II:1 (F, 2.5 m)	c.753_754del (p.Val253Alafs*44)	c.753_754del (p.Val253Alafs*44)	Yes	Yes	NA	NA	Yes	Yes	Yes	Yes	–	NA	NA	NA	NA	NA	^20^
Family B (Fetus)	c.753_754del (p.Val253Alafs*44)	c.753_754del (p.Val253Alafs*44)	NA	NA	NA	NA	NA	NA	NA	NA	–	NA	NA	NA	NA	NA	^12^
EG-2 V-1 (F, 21 y)*	c.807dup (p.Gly270Trpfs*28)	c.807dup (p.Gly270Trpfs*28)	Yes	Yes	No	Yes	Yes	Yes	Yes	Yes	–	No	NA	No	No	No	^5^
EG-2 V-2 (M, 16 y)*			Yes	Yes	No	Yes	Yes	Yes	Yes	Yes	Yes	No	Yes	No	No	NA	^5^
Family 1 IV:3 (F, 1 m)	c.1010_1011del (p.His337Leufs*60)	c.1010_1011del (p.His337Leufs*60)	Yes	Yes	NA	NA	Yes	Yes	NA	Yes	–	NA	NA	NA	NA	NA	^20^
UPN-0315 (Fetus)	c.1180C>G (p.Pro395Ala)	c.1180C>G (p.Pro395Ala)	NA	NA	NA	NA	Yes	NA	NA	NA	–	NA	NA	NA	NA	NA	^15^
MPS036 (NA, NA)	c.1292_1311del (p.Leu431Hisfs*22)	c.1292_1311del (p.Leu431Hisfs*22)	NA	NA	NA	NA	NA	NA	NA	NA	NA	NA	NA	NA	NA	NA	^6^
EG-5 V-1 (M, 14 y)*	c.1249G>C (p.Glu417Gln)	c.1249G>C (p.Glu417Gln)	Yes	Yes	No	Yes	Yes	Yes	No	Yes	Yes	No	Yes	No	No	NA	^5^
EG-5 V-4 (F, 7 y)*			Yes	Yes	No	Yes	Yes	Yes	No	Yes	–	No	No	No	No	NA	^5^
UPN-0315 (F, 5 m)	c.1366_1367del (p.His457Leufs*2)	c.1366_1367del (p.His457Leufs*2)	NA	NA	NA	NA	NA	NA	NA	NA	–	NA	NA	NA	NA	NA	^15^
Frequency of features (Yes/Yes+No)			86.8% (33/38)	94.6% (35/37)	30.8% (4/13)	87.5% (21/24)	100% (38/38)	100% (26/26)	80.8% (21/26)	100% (19/19)	100%** (11/11)	58.8% (10/17)	75.0% (9/12)	64.3% (9/14)	9.1% (1/11)	14.3% (1/7)	
*P* value			0.3422	0.026*	<0.001***	0.412	0.002**	0.010*	0.950	0.030*	0.104	0.015*	0.573	0.096	<0.001***	<0.001***	

^*^Siblings; #, pregnancy was terminated at 15 weeks of gestation.

**The prevalence of cryptorchidism was calculated based on male patients evaluated for this condition.

*F* female, *M* male, *d* days, *m* months, *y* years.

Pathogenic variants in genes of the AChR pathway (*CHRNA1*, *CHRNB1*, *CHRND*, *CHRNG* and *RAPSN*) are associated with a spectrum of fetal akinesia deformation sequence, characterized by restricted intrauterine movement, growth retardation, fetal hydrops, micrognathia and multiple joint contractures with or without pterygia^24^. Among these, *CHRNG* encodes the γ-subunit of the embryonic nicotinic AChR and plays key roles not only in neuromuscular transmission but also in neuromuscular junction development during the fetal period^25^.

*CHRNG* variants underlie both the nonlethal Escobar phenotype and the lethal form of multiple pterygium syndrome (MPS), and have also been reported in fetal akinesia deformation sequence without pterygia. In our literature review (Table 1), pterygia, facial dysmorphism, high-arched palate, arachnodactyly/camptodactyly, kyphosis/scoliosis and short stature were frequently observed in patients with pathogenic *CHRNG* variants. Although no previously reported patients carried the same variant combination as our patient, individuals harboring c.428C>G on one allele generally did not develop pterygia and tended to show arthrogryposis multiplex, arachnodactyly/camptodactyly and short stature. Compared with reported individuals compound heterozygous for c.428C>G and c.753_754del (p.Val253Alafs44), as well as an individual compound heterozygous for c.428C>G and c.255T>A (p.Trp85*), the present patient is expected to exhibit a broadly similar clinical phenotype.

In our literature search, we did not identify any case reports of individuals harboring the c.2T>C (p.Met1?) variant in *CHRNG* (Table 1). However, start-loss variants such as c.2T>C (p.Met1?) in other genes have been associated with severe disease in hemizygous individuals or compound heterozygotes^26–30^ (Supplementary Data 3). Taken together, c.2T>C (p.Met1?) is likely to have a substantial functional impact on CHRNG.

Moreover, we performed an in silico analysis of translation initiation using NetStart 2.0, a deep-learning-based predictor of translation initiation sites^31^. This analysis predicted a downstream in-frame alternative initiation codon with a high confidence score (0.993), suggesting that translation may initiate downstream of the canonical start site and produce an N-terminally truncated CHRNG protein lacking approximately 33 amino acids. Therefore, the c.2T>C (p.Met1?) variant should not be assumed to be a nonsense-equivalent null allele.

Nevertheless, the N-terminal extracellular domain of the γ subunit is essential for proper folding and assembly of the embryonal AChR and contributes to formation of the α–γ agonist-binding interface^32,33^. Accordingly, loss of the N-terminal region is expected to impair receptor assembly and/or activation efficiency, even if a truncated protein is produced.

Consistent with this interpretation, our patient’s clinical phenotype was milder than that observed in individuals harboring biallelic truncating *CHRNG* variants, yet sufficiently severe to produce fetal akinesia-related manifestations such as congenital contractures and neonatal asphyxia^4^. Taken together, these findings suggest that c.2T>C p.(Met1?) represents a hypomorphic loss-of-function allele, leading to partial but functionally substantial reduction of CHRNG activity. Because these conclusions are based on in silico predictions, functional analyses at the protein level will be necessary to confirm their biological relevance.

Vogt et al.^6^ reported that nearly all patients with MPS carrying *CHRNG* variants exhibited pterygia, suggesting a strong association between this genetic defect and pterygia formation. By contrast, our patient did not display pterygia and had an overall milder course, with manifestations largely limited to congenital arthrogryposis multiplex. This observation supports a broader *CHRNG*-related phenotypic spectrum and indicates that congenital contractures in the absence of pterygia may still reflect an underlying fetal akinesia deformation sequence, particularly given the severe neonatal asphyxia in this case.

Short stature is also frequently observed in Escobar syndrome, with more than 55% of affected individuals reportedly exhibiting this feature^34^. In several studies, short stature has been associated with decreased IGF-1 levels, which play important roles in fetal skeletal development^35^. In murine models, acetylcholine deficiency reduces IGF-1 levels and has been proposed to modulate IGF-1 secretion^36^. IGF-1 promotes development, growth, differentiation and maintenance of bone and muscle strength^37^. However, this patient had a normal IGF-1 level, and her growth impairment appeared attenuated.

The pleiotropic effects of *CHRNG* variants should also be considered. Associations with congenital diaphragmatic muscle weakness, pulmonary hypoplasia and intraspinal abnormalities such as syringomyelia or tethered cord syndrome have been reported. These potential complications warrant attention when planning surgical procedures or rehabilitation programs.

In conclusion, compound heterozygosity for *CHRNG* variants NM_005199.5:c.[2T>C];[428C>G], p.[(Met1?)];[(Pro143Arg)] may present with neonatal asphyxia, congenital contractures, low-frequency hearing loss and short stature while sparing cognition, even in the absence of pterygia, thereby broadening the recognized Escobar spectrum. Recognition of this attenuated phenotype supports accurate counseling and appropriate longitudinal management.

## HGV Database

The relevant data from this Data Report are hosted at the Human Genome Variation Database at 10.6084/m9.figshare.hgv.3598 and 10.6084/m9.figshare.hgv.3601.

## Supplementary information

Supplementary Data 1. Laboratory data at age of 10 years and 3 months.

Supplementary Data 2. The variants detected in *CHRNG* gene.

Supplementary Data 3. An example of start-loss variants.
