# Supplementary material for: Non-pterygium Escobar syndrome from compound-heterozygous CHRNG variants: genotype–phenotype insights
Source: Hum Genome Var. 2026 Mar 14;13:8. doi: 10.1038/s41439-026-00340-8 (PMC13000176; doi:10.1038/s41439-026-00340-8)
Supplement: Supplementary file 1 — Supplementary Data 1. Laboratory data at age of 10 years and 3 months. [file 41439_2026_340_MOESM1_ESM.docx]

Supplementary Data 1. Laboratory data at age of 10 years and 3 months

| WBC | 5300 | /μL | TP | 7.2 | g/dL | TSH | 0.88 | μIU/mL |
| --- | --- | --- | --- | --- | --- | --- | --- | --- |
| Ly | 52.0 | % | Alb | 4.5 | g/dL | F-T3 | 4.10 | pg/mL |
| Neut | 43.1 | % | T-Bil | 0.6 | mg/dL | F-T4 | 0.93 | ng/dL |
| RBC | 473 | X10^4^/μL | AST | 18 | IU/L | GH | 0.95 | ng/mL |
| Hb | 13.8 | g/dL | ALT | 10 | IU/L | LH | 4.1 | mIU/mL |
| Plt | 26.6 | X10^4^/μL | LDH | 204 | IU/L | FSH | 6.6 | mIU/mL |
|  |  |  | γ-GTP | 11 | IU/L | IGF-1 | 347 | ng/mL |
|  |  |  | CK | 84 | IU/L | HCG | <1.0 | mIU/mL |
|  |  |  | BUN | 9.3 | mg/dL | Progesterone | 0.1 | ng/mL |
|  |  |  | Cr | 0.32 | mg/dL |  |  |  |
|  |  |  | Na | 140 | mEq/L |  |  |  |
|  |  |  | K | 4.7 | mEq/L |  |  |  |
|  |  |  | Cl | 108 | mEq/L |  |  |  |
|  |  |  | Ca | 10.0 | mg/dL |  |  |  |
|  |  |  | CRP | 0.01 | mg/dL |  |  |  |

WBC: white blood cell, Ly: lymphocyte, Neut: neutrophil, RBC: red blood cell, Hb: hemoglobin, Plt: platelets, TP: total protein, Alb: albumin, T-Bil: total bilirubin, AST: aspartate aminotransferase, ALT: alanine aminotransferase, LDH: lactate dehydrogenase, γ-GTP: γ-glutamyl transpeptidase, CK: creatine kinase, BUN: blood urea nitrogen, Cre: creatinine, TSH: thyroid stimulating hormone, T3: triiodothyronine, T4: thyroxine, GH: growth hormone, LH: luteinizing hormone, FSH: follicle stimulating hormone, IGF-1: insulin-like growth factor 1, HCG: human chorionic gonadotropin
