# Supplementary material for: Non-pterygium Escobar syndrome from compound-heterozygous CHRNG variants: genotype–phenotype insights
Source: Hum Genome Var. 2026 Mar 14;13:8. doi: 10.1038/s41439-026-00340-8 (PMC13000176; doi:10.1038/s41439-026-00340-8)
Supplement: Supplementary file 2 — Supplementary Data 2. The variants detected in CHRNG gene. [file 41439_2026_340_MOESM2_ESM.docx]

Supplementary Data 2. The variants detected in *CHRNG* gene

| No. | Nucleic acid | Amino acid | Location | ClinVar | Polyphen-2 (Score) | Allele frequency | | References |
| --- | --- | --- | --- | --- | --- | --- | --- | --- |
|  |  |  |  |  |  | jMorp | gnomAD |  |
| 1 | c.2T>C | p.Met1? | Exon 1 | LP | B (0.010) | 0.000008 | NR | This study |
| 2 | c.13C>T | p.Gln5* | Exon 1 | LP | NA | NR | NR | [5] |
| 3 | c.55G>A | p.Gly19Arg | Exon 1 | NR | PoD (0.923) | NR | NR | [6] |
| 4 | c.56-1G>A | unknown | Intron 1 | P | NA | NR | 0.000026 | [6] |
| 5 | c.117dup | p.Asn40Glnfs*96 | Exon 2 | P | NA | NR | 0.000086 | [13] |
| 6 | c.136C>T | p.Arg46* | Exon 2 | P | NA | NR | 0.000026 | [4] |
| 7 | c.202C>T | p.Arg68* | Exon 3 | P/LP | NA | 0.000025 | 0.000026 | [14], [17] |
| 8 | c.241C>T | p.Gln81* | Exon 4 | P | NA | NR | NR | [18] |
| 9 | c.255T>A | p.Try85* | Exon 4 | NR | NA | 0.000025 | NR | [11] |
| 10 | c.256C>T | p.Arg86Cys | Exon 4 | P/LP | PrD (1.000) | 0.000212 | 0.000007 | [5], [18], |
| 11 | c.292_300dup | p.Leu100_Arg101insTrpValLeu | Exon 4 | LP | NA | NR | 0.000007 | [5], [14], [17], |
| 12 | c.299T>G | p.Leu100Arg | Exon 4 | NR | PoD (0.602) | NR | NR | [17] |
| 13 | c.320T>G | p.Val107Gly | Exon 4 | LP | PrD (0.999) | NR | NR | [4] |
| 14 | c.351-9T>C | unknown | Intron 4 | C | NA | NR | 0.000007 | [4] |
| 15 | c.388del | p.Val130Cysfs*53 | Exon 5 | NR | NA | NR | NR | [6] |
| 16 | c.397del | p.Ser133Profs*50 | Exon 5 | P | NA | NR | 0.000033 | [6] |
| 17 | c.401_402del | p.Pro134Argfs*34 | Exon 5 | P | NA | 0.000024 | 0.000099 | [4], [14], [20] |
| 18 | c.428C>G | p.Pro143Arg | Exon 5 | P | PrD (1.000) | 0.000408 | 0.000007 | This study, [11], [22] |
| 19 | c.459dup | p.Val154Serfs*24 | Exon 5 | P | NA | NR | 0.000453 | [4], [14], [17], [21], [23], [19] |
| 20 | c.482G>A | p.Trp161* | Exon 5 | P | NA | NR | NR | [5] |
| 21 | c.518dup | p.Tyr173* | Exon 6 | P | NA | NR | NR | [12] |
| 22 | c.639_643del | p.Lys214Alafs*82 | Exon 7 | NR | NA | NR | NR | [14] |
| 23 | c.715C>T | p.Arg239Cys | Exon 7 | P | PrD (1.000) | NR | 0.000020 | [5], [16], [17], [18] |
| 24 | c.753_754del | p.Val253Alafs*44 | Exon 7 | P | NA | 0.000188 | 0.000256 | [4], [11], [12], [14], [16], [17], [18], [20], [22] |
| 25 | c.794T>G | p.Leu265Arg | Exon 7 | LP | PrD (1.000) | NR | NR | [23] |
| 26 | c.807dup | p.Gly270Trpfs*28 | Exon 8 | P | NA | NR | NR | [5] |
| 27 | c.1010_1011del | p.His337Leufs*60 | Exon 9 | LP | NA | NR | NR | [20] |
| 28 | c.1132_1136dup | p.Gly380Profs*39 | Exon 10 | NR | NA | NR | NR | [6] |
| 29 | c.1180C>G | p.Pro394Ala | Exon 10 | U | PoD (0.484) | NR | 0.000026 | [15] |
| 30 | c.1249G>C | p.Glu417Gln | Exon 10 | NR | B (0.035) | NR | NR | [5] |
| 31 | c.1292_1311del | p.Leu431Hisfs*22 | Exon 11 | P | NA | NR | NR | [6] |
| 32 | c.1366_1367del | p.His457Leufs*2 | Exon 11 | U | NA | 0.000041 | 0.000007 | [15] |
| 33 | c.1408C>T | p.Arg470* | Exon 12 | LP | NA | NR | NR | [5] |

NA: not available, NR: not registered, P: pathogenic, LP: likely pathogenic, C: Conflicting classifications of pathogenicity, U: uncertain significance, B: benign, PrD: probably damaging, PoD: possibly damaging

jMorp: Japanese Multi Omics Reference Panel
