# Supplementary material for: Non-pterygium Escobar syndrome from compound-heterozygous CHRNG variants: genotype–phenotype insights
Source: Hum Genome Var. 2026 Mar 14;13:8. doi: 10.1038/s41439-026-00340-8 (PMC13000176; doi:10.1038/s41439-026-00340-8)
Supplement: Supplementary file 3 — Supplementary Data 3. An example of start-loss variants. [file 41439_2026_340_MOESM3_ESM.docx]

Supplementary Data 3. The example of start-loss variants

| Diseases | Genes | Location | Variants | | Classification | | References |
| --- | --- | --- | --- | --- | --- | --- | --- |
|  |  |  | Nucleic acid | Amino acid | ClinVar | ACMG |  |
| Cystic fibrosis | *CFTR* | 7q31.2 | c.2T>C | p.M1? | Pathogenic | PVS1, PM2, PP5 | [26] |
| Fanconi anemia | *FANCA* | 16q24.3 | c.2T>C | p.M1? | Pathogenic/Likely pathogenic | Not specified | [27] |
| Fabry | *GLA* | Xq22.1 | c.2T>C | p.M1? | Pathogenic | Not specified | [28], [29] |
| Juvenile polyposis | *BMPR1A* | 10q23.2 | c.1A>C | p.M1? | Pathogenic/Likely pathogenic | Not specified | [30] |

PVS: Very strong evidence of pathogenicity, PM: Moderate evidence of pathogenicity, PP: Supporting evidence of pathogenicity
